# Immune responses in cardiac repair and regeneration: a comparative point of view

**DOI:** 10.1007/s00018-018-2995-5

**Published:** 2018-12-21

**Authors:** Shih-Lei Lai, Rubén Marín-Juez, Didier Y. R. Stainier

**Affiliations:** 1 Department of Developmental Genetics, Max Planck Institute for Heart and Lung Research, Bad Nauheim, Germany; 2 Institute of Biomedical Sciences, Academia Sinica, Taipei, Taiwan

**Keywords:** Myocardial infarction, Macrophages, Neutrophils, Scar formation, Scar resolution, Neonatal mice, Salamander, Zebrafish, Medaka, Comparative analysis

## Abstract

Immediately after cardiac injury, the immune system plays major roles in repair and regeneration as it becomes involved in a number of processes including damage-associated signaling, inflammation, revascularization, cardiomyocyte dedifferentiation and replenishment, and fibrotic scar formation/resolution. Recent studies have revealed that different immune responses occur in the various experimental models capable or incapable of cardiac regeneration, and that harnessing these immune responses might improve cardiac repair. In light of this concept, this review analyzes current knowledge about the immune responses to cardiac injury from a comparative perspective. Insights gained from such comparative analyses may provide ways to modulate the immune response as a potential therapeutic strategy for cardiac disease.

## Introduction/Background

Heart failure is a major cause of morbidity and mortality, in part because of the inability of the human heart to replenish lost muscle tissue from cardiomyopathies including myocardial infarction (MI). Instead, fibrotic scar forms during the repair process, compromises cardiac function, and eventually leads to adverse remodeling and failure. Interestingly, recent studies have reported that adult cardiac muscle cells (cardiomyocytes, CMs) retain some capacity to divide in both mice [7, 21, 44, 64, 92, 94, 108, 131, 125] and humans [5, 6, 77], thus raising the possibility of promoting endogenous cardiac regeneration in patients.

Regenerative and non-regenerative models provide opportunities for comparative analyses to gain knowledge regarding cardiac regeneration, as well as to develop new therapeutic strategies for human cardiac disease [17, 99, 104]. Interestingly, comparative studies between neonatal and adult mice [3, 59], and between phylogenetically related species such as zebrafish (Danio Rerio) and medaka (Oryzias latipes) [58] have suggested that the capacity for regeneration does not solely rely on genetic makeup, environmental conditions (e.g., hypoxia), or evolutionary complexity; instead, the type and extent of the immune responses to cardiac injury seem to be a major difference between these regenerative and non-regenerative models [3, 58, 59], and may largely influence the recovery post experimental MI, as well as clinical prognosis [30, 96].

### Injury models

**Fig. 1:**
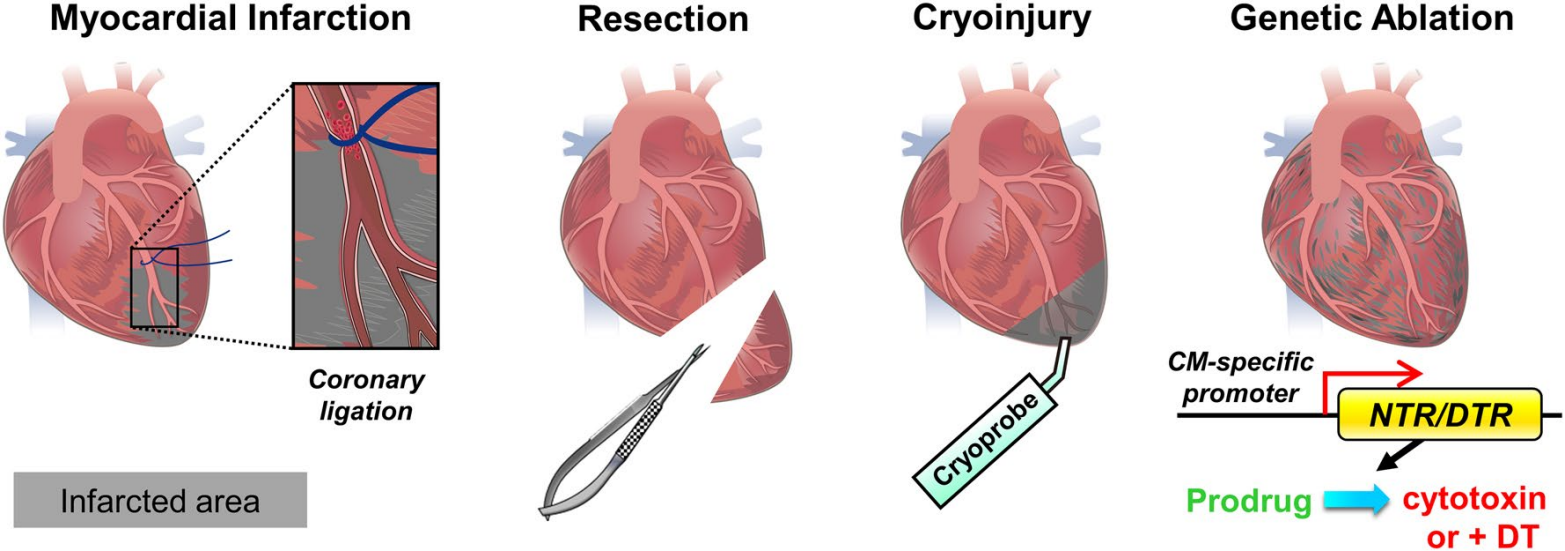
Cardiac injury models. Illustration of myocardial infarction (MI), resection, cryoinjury and genetic ablation of cardiomyocytes (CMs). MI is induced by surgically ligating the left anterior descending coronary artery, leading to tissue death downstream of the ligature. Resection is used to remove part of the ventricle. Cryoinjury is used to cauterize part of the ventricle with a cryoprobe. Genetic ablation is achieved by driving CM-specific Nitroreductase (NTR) expression, which in turn converts a prodrug into a cytotoxic product leading to CM death; alternatively, CM-specific expression of the Diphtheria toxin receptor (DTR) will render the CMs susceptible to diphtheria toxin (DT)-induced cell death

To study cardiac repair and regeneration, various injury models have been established to induce myocardial lesion, including myocardial infarction (MI), resection, cryoinjury and genetic ablation (Fig. 1). Experimental MI is induced by ligating the left anterior descending coronary artery to cut off blood flow, leading to ischemic cell death of the downstream tissue. This method is usually performed in rodents and larger mammals and best mimics the pathological condition in humans (Fig. 1) [26, 40, 94]. In the ischemic reperfusion (I/R) MI model, the vessel ligature is released after 30 min, mimicking the pathophysiology of clinical reperfusion. In contrast to permanent MI, reperfusion salvages ischemic myocardium but paradoxically causes injury due to reactive oxidative species (ROS) production and altered inflammation [39]. Since vessel ligation is more practical in larger animals with a distinct coronary system, other injury methods have been established. For example, resection can be performed on almost all animals by surgically removing a part of the ventricle (Fig. 1). Although resection efficiently induces tissue loss, unlike MI, it induces less cell necrosis and fibrotic scar formation in the remaining tissue [22, 93, 95). Cryoinjury is another frequently adopted method and it consists of cauterizing the ventricle with a metal probe (cryoprobe) equilibrated in liquid nitrogen (Fig. 1). Similar to MI, cryoinjury results in cell necrosis alongside healthy tissue and formation of a prominent fibrotic scar, which also closely resembles the pathophysiological condition in humans [12, 35, 106, 115]. Alternatively, genetic ablation can be achieved by expressing bacterial Nitroreductase (NTR) or Diphtheria toxin receptor (DTR) specifically in CMs (Fig. 1). NTR catalyzes the reduction of innocuous prodrugs such as Metronidazole (Mtz) into a cytotoxic product leading to cell death [19, 20]. Expression of DTR in CMs makes them susceptible to Diphtheria toxin-induced cell death [101]. Although genetic ablation is a convenient way to induce CM death without any surgery, the resulting fibrotic scar is difficult to quantify and compare [59].

### Animal models

With respect to animal models, general capacity for tissue regeneration seems to inversely correlate with evolutionary complexity across the animal kingdom [124, 137]. Unlike mammals, some vertebrates are capable of endogenous heart regeneration in adulthood, including certain fish and amphibians [124]. Zebrafish are a favored model as they exhibit a remarkable regenerative capacity after various cardiac insults [12, 35, 95, 106, 139]. Recently, another fresh water teleost, medaka (*Oryzias latipes*), has been reported to lack revascularization and CM proliferation after cardiac injury, subsequently displaying excessive fibrosis and an unresolved scar [47]. Direct comparison between zebrafish and medaka represents a unique platform to identify and investigate mechanisms underlying cardiac regeneration [58]. Another recent study reported differential regenerative capacity in Mexican cavefish (Astyanax mexicanus) living in surface dwellings versus those living in caves, and revealed a complex interplay between CM proliferation and scar resolution [114]. QTL analysis further revealed 3 loci modulating heart regeneration in cavefish and modern genomic and genetic approaches should lead to their identification in the near future.

In terms of the mammalian models, newborn mice retain a certain level of regenerative capacity in response to cardiac injury [40, 93, 115]. Despite this regenerative capacity, variable amounts of residual fibrotic tissues remain in neonatal hearts after recovery, depending on the injury model used and the extent of injury [55, 107]. This regenerative capacity is mostly lost by 7 days after birth, and adult mice exhibit minimal CM replenishment, unresolved scars and contractile dysfunction after cardiac injury [40, 93, 115]. Comparative studies between neonatal and adult mice have already been very informative [3, 59, 73], and they are like to remain a powerful approach.

### Inflammatory signaling and cellular contribution

The immune response post MI can be temporally divided into the pro-inflammatory phase and the inflammatory resolution/reparative phase, involving components of both the innate and adaptive immune systems. Inflammation after cardiac injury is mainly triggered by molecules released from necrotic cells and it is programmed to be resolved when the cell debris is cleared. This process takes place in both regenerative and non-regenerative models, yet profound differences can be observed which lead to scar resolution and tissue replenishment in the former, and scar maturation and tissue remodeling in the latter. These differences are due at least in part from the differential response of cardiac resident cells and recruited immune cells in regenerative versus non-regenerative models. Of note, prolonged and unresolved inflammation seems to enhance the fibrotic response and worsen functional recovery during the recovery phase [30]. This review tries to distill our current understanding of the roles of the immune response during cardiac repair with a focus on inflammation. We also discuss the differential immune response in regenerative versus non-regenerative models, as well as potential strategies to modulate the immune response to improve cardiac recovery.

## Inflammation

After MI, tissue damage rapidly triggers the response of the immune system. Immune cells are initially recruited to the injured tissue, clear debris and dead cells, and degrade the extracellular matrix [29]. Later, programmed resolution of inflammation allows the recruitment and activation of myofibroblasts for extracellular matrix (ECM) deposition and vascular cells to stabilize the new vessels [29]. Inflammation is both essential and deleterious for cardiac repair and regeneration, depending on its precise spatial and temporal regulation. Inflammation is required for cardiac regeneration post injury [3, 45, 58, 59]. In addition, acute inflammation initiates a reparative response in neonatal mouse hearts [36] and can precondition the heart for effective regeneration in zebrafish [23]. On the other hand, studies in mice suggest that inflammation extends tissue damage post MI, while minimizing inflammation reduces the infarct size and adverse remodeling [2, 86, 120]). In light of the importance and complexity of the inflammatory response, we discuss in detail the tight regulation of inflammation initiation and resolution during cardiac healing.

### Triggers of inflammation: DAMPs, complements, reactive oxygen species (ROS) and TLR signaling

**Fig. 2:**
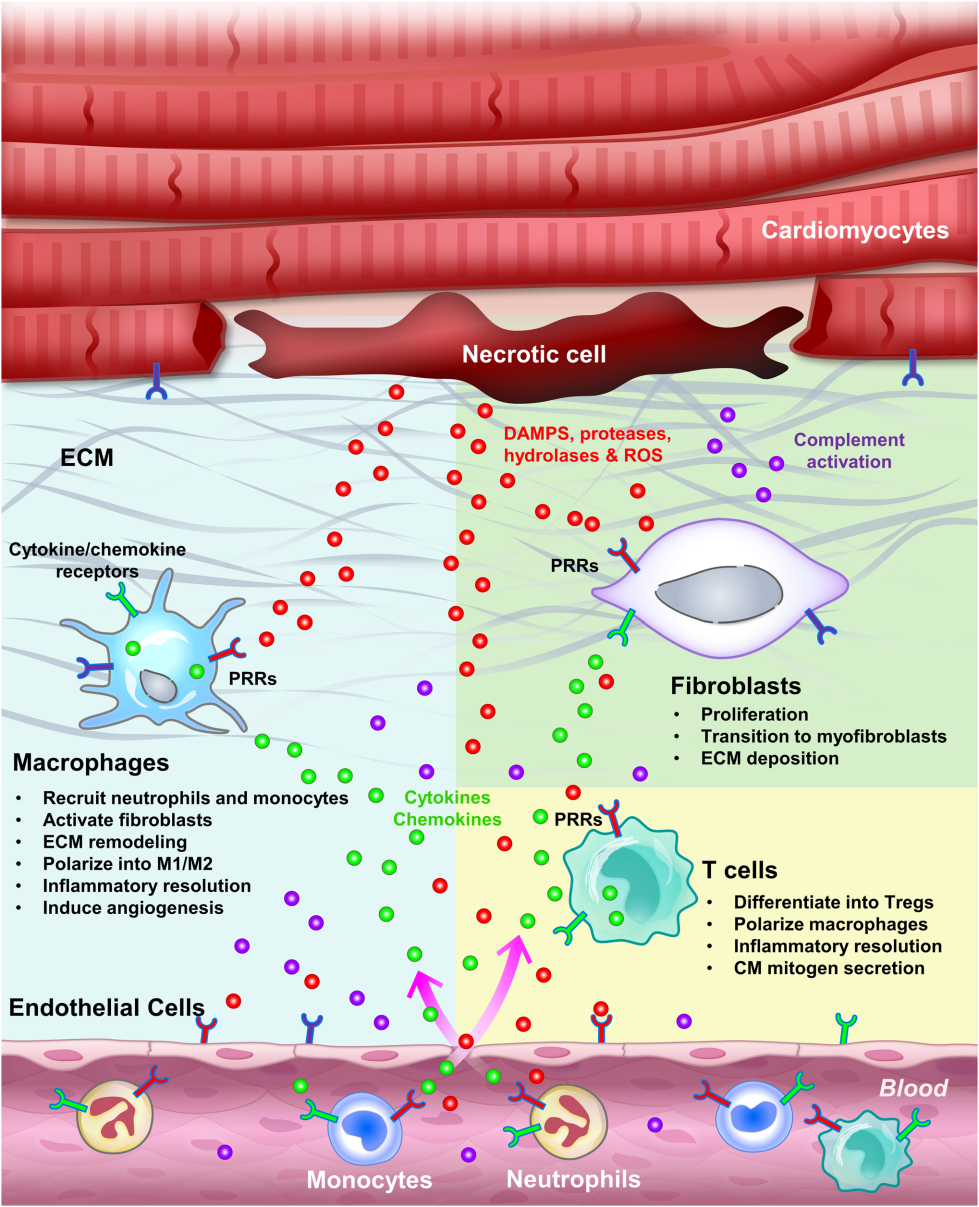
Inflammation induced by cardiac injury. Sterile inflammation can be triggered by various components released by necrotic cells, including DAMPs, proteases, hydrolases and mitochondrial ROS. DAMPs directly activate PRRs on surveillant cells, including tissue macrophages, circulating monocytes and neutrophils, as well as on resident cells, including endothelial cells, fibroblasts and CMs. Proteases, hydrolases and ROS activate the complement system as well as inflammasomes, and degrade the ECM, altogether further propagating the inflammatory response. Activated tissue resident macrophages secrete cytokines to attract monocytes and neutrophils, activate endothelial cells to promote cell adhesion and permeability, and remodel the ECM. Infiltrating monocytes and neutrophils clear cell debris by phagocytosis and help terminate the initial insult. After wound clearance, myofibroblasts secrete ECM to help prevent the injured heart from rupturing. Differentiated Tregs tune down the inflammation by secreting anti-inflammatory cytokines, in parallel with M1–M2 macrophage polarization and programmed neutrophil apoptosis. Inflammation initiation, propagation and resolution can occur in both regenerative and non-regenerative models. However, in the regenerative models, these processes seem to facilitate CM dedifferentiation and proliferation and scar resolution by mechanisms yet to be determined

Pathogen-associated innate immune responses are well described and are canonically induced through pattern recognition receptors (PRRs), including Toll-like receptors (TLRs) and the receptor for advanced glycation end products (RAGE), which are expressed in both recruited leukocytes and tissue resident cells [8, 14, 119]. In contrast, sterile inflammation, which occurs during MI, is triggered by endogenous molecules known as damage-associated molecular patterns (DAMPs) or alarmins [16]. Similar to how pathogens induce inflammation, DAMPs also trigger the innate immune response and inflammation by binding to PRRs (Fig. 2) [8, 14, 119]. On the other hand, release of cellular components such as proteases, hydrolases, and mitochondrial ROS also activates the complement system and generates further DAMPs, including fragmented ECM, to initiate and propagate the inflammatory response [56]. Activation of PRRs on surveillant immune cells including resident macrophages and circulating monocytes, as well as resident cells, further induces the expression and secretion of various inflammatory cytokines and chemokines, and propagates the inflammatory response [8, 14, 119]. This sterile inflammatory response seems to be essential to clear the initial insult (necrotic cells) and activate reparative responses such as CM dedifferentiation and proliferation, but at the same time might lead to extended injury if not resolved in a timely manner.

#### DAMPs

High-mobility group B1 (HMGB1) is the best characterized MI-induced DAMP. In the mouse MI model, HMGB1 is an important chromatin-binding protein released by necrotic cells and/or secreted by macrophages [9]. HMGB1 promotes the maturation and migration of immune cells by interacting with PRRs, such as TLR2/4 and RAGE [54, 111]. Upon release or secretion, HMGB1 facilitates tissue repair and healing by promoting the switch of macrophages to a tissue-healing phenotype, the activation and proliferation of stem cells, and neoangiogenesis [10]. In addition, HMGB proteins also function as universal sentinels for nucleic acid-mediated innate immune responses. Extracellular DNA and RNA released by necrotic cells bind to macrophage TLRs and activate the secretion of pro-inflammatory cytokines, including IL-6, IL-12, and Tumor Necrosis Factor alpha (TNFα) [48, 134]. Released nucleic acids play dual roles upon cardiac insult. On the one hand, extracellular RNA released during both I/R injury and MI promotes myocardial inflammation and reducing serum RNA levels by RNase administration conferred cardiac protection against I/R injury [15, 63]. Conversely, extracellular DNA activates TLR9 signaling and promotes the proliferation and differentiation of cardiac fibroblasts into myofibroblasts. Myofibroblasts in turn promote ECM deposition and prevent cardiac rupture after MI in mice [84]. Interestingly, administration of the TLR9 agonist CpG-ODN prior to I/R injury induced preconditioning and attenuated myocardial injury via upregulation of the anti-inflammatory cytokine IL-10 [11, 66]. Furthermore, administration of the TLR3 agonist poly I:C after cardiac injury promoted regeneration in the non-regenerative medaka [58]. The roles of TLR signaling in cardiac healing will be discussed in detail in a later section.

The ECM provides mechanical support and maintains the structural integrity of the heart. During the inflammatory phase of MI in mice, high matrix metalloproteinase (MMP) activity from necrotic cells, neutrophils and macrophages degrades the cardiac ECM. The resulting ECM fragments play active roles in inflammatory propagation/modulation, signal transduction and mechanical remodeling during cardiac repair [25, 31, 129]. Interestingly, a common transition from the early fibrin-enriched ECM environment to the late collagen-based mature scar occurs after cardiac injury in both regenerative and non-regenerative models [31, 35]. The transient fibrin-based ECM modulates inflammation by regulating leukocyte engagement via integrin receptors, influencing immune cell behavior, and stimulating macrophage chemokine secretion through TLRs [18, 27, 112]. It may also serve as a scaffold for migrating inflammatory cells and as a support for proliferating endothelial cells and fibroblasts [31]. Accordingly with their critical roles, ECM synthesis genes are amongst the most upregulated ones post injury in both the neonatal mouse heart [40] and the zebrafish heart [58, 61, 71]. The ECM components fibronectin and tenascin-C are essential for cell cycle re-entry and proliferation of CMs during zebrafish and newt heart regeneration [71, 127]. Subsequently, the mature collagen-based scar resolves without causing adverse remodeling in regenerative models [35, 93]. The cellular and molecular mechanisms of scar resolution are still unknown. From a comparative perspective, it may be extremely valuable to determine the potential differences in ECM composition and dynamic changes in regenerative and non-regenerative models, and further investigate how the ECM potentially modulates cardiac healing. As a recent example, AGRIN was identified as a cardiac ECM component enriched in neonatal compared to adult mice, and it is required for the full reparative capacity in neonates [4]. Moreover, a single administration of AGRIN could promote regenerative capacity in adult mice after MI [4].

#### The complement system

The complement system is another critical component of innate immunity, and it functions as the first defense to eliminate pathogens by marking them for phagocytosis or directly lysing them via the assembly of the membrane attack complex [13, 100]. In addition, the complement system triggers inflammation via C3a and C5a fragments (anaphylatoxins) [13, 100]. These fragments facilitate neutrophil recruitment through activation of the endothelium to increase vessel permeability and leukocyte adhesion [13, 100]. Moreover, C3a and C5a fragments might induce fibrotic repair after MI by facilitating and modulating cardiac pluripotent/progenitor cell (CPC) differentiation towards fibroblasts/myofibroblasts. On the other hand, the complement system is also involved in tissue repair and regeneration [100, 116]. In the heart, the complement receptor gene *C5aR1* is activated in CMs and endothelial cells after cardiac resection in regenerative models including axolotl, zebrafish and neonatal mice [82]. Inhibition of C5aR1 significantly attenuated the activation of CM proliferation in all three species, suggesting that C5aR1 mediates an evolutionarily conserved response to cardiac injury [82]. Of note, differences in macrophage infiltration of the injured zone between *C5aR1* WT and knockout mice were observed, despite the fact that *C5aR1* expression was not colocalized with macrophage markers [82]. These findings suggest a role for C5aR1 in non-myeloid cells during cardiac regeneration, but the detailed mechanisms remain unclear. Clearly, a lot more work needs to be done on the complement system to understand its role in modulating the immune response and other aspects of the regenerative process.

#### Reactive oxygen species (ROS)

ROS have been implicated as the cue to promote immune cell infiltration into sites of injury [78, 83]. ROS released from mitochondria of necrotic cells or secreted by neutrophils trigger the inflammatory response through direct activation of the inflammasome in cardiac resident cells including fibroblasts [51] and mast cells [32]. Inflammasome activation leads to the maturation and secretion of the pro-inflammatory cytokines Interleukin 1β (IL-1β) and Interleukin 18 (IL-18) [68].

Excessive generation of ROS during reperfusion induces inflammation and extended myocardial injury [79]. Consistently, ROS production in the mouse heart during the first week of birth causes DNA damage and cell cycle arrest in CMs, and may account for the decreased regenerative capacity in adults [97]. Furthermore, chronic hypoxia minimizes ROS production and induces CM proliferation following MI, resulting in significant recovery of left ventricle (LV) systolic function [53, 81]. These findings support the hypothesis that reduction in ROS and oxidative DNA damage favors CM cycling.

Besides their harmful role when present at high levels during early reperfusion, minimal ROS levels during ischaemia and/or at reperfusion are critical for the redox signaling of cardioprotection, which reduces the extent of infarct [79]. In the context of regeneration, hydrogen peroxide, a major ROS, initiates inflammation by rapid recruitment of leukocytes to the wound in zebrafish [83], and promotes CM proliferation and cardiac repair [37]. Nevertheless, how redox signaling is balanced during heart regeneration and if it can be better modulated in non-regenerative models remain elusive. These findings underscore the importance of redox signaling during cardiac healing and call for further investigation.

#### TLR signaling

As the most prominent members of PRRs, TLRs belong to a family of transmembrane receptors responding to various DAMPs, resulting in the activation of pro-inflammatory cytokine and chemokine genes [96]. Functional studies indicate that TLRs are important mediators of the post-infarction inflammatory reaction, whereby the loss of function of some TLRs seems to be beneficial for cardiac healing after MI [2, 86, 120].

Despite a general acknowledgment of their role in triggering inflammatory responses, a detailed understanding of TLR signaling in cardiac regeneration remains elusive. TLR signaling seems to trigger CM proliferation during regeneration. Administration of zymosan or lipopolysaccharides (LPS), TLR2 and TLR4 agonists, respectively, preconditions CMs for cell cycle re-entry in zebrafish [23], and induced CM proliferation in the neonatal mouse heart [36]. In zebrafish, LPS administration also triggers the expression of the retinoic acid synthesizing enzyme Aldh1a2, which has been shown to be required for CM proliferation during heart regeneration [52]. Furthermore, in non-regenerative medaka, IP injection of the TLR3 agonist poly I:C promotes macrophage recruitment, revascularization, and CM proliferation [58]. Altogether, these data suggest that although damage signals appear to trigger inflammation and lead to extended infarction in adult mammalian MI models, differential responsiveness of TLR signaling to particular stimuli might induce protection or regeneration. To better understand how differential TLR responsiveness modulates cardiac healing, thorough examination of transcriptomic and cytokine responses upon various TLR ligand stimulation should be performed in both regenerative and non-regenerative models. The detailed function of each TLR should also be determined to reveal the mechanisms of preconditioning and how each TLR contributes to the overall inflammatory response post cardiac injury.

### Cellular components involved in inflammation

Damage signals trigger inflammation in both resident cells (including endothelial cells, fibroblasts, mast cells, tissue resident macrophages, CMs, and epicardial cells), as well as in recruited cells (including neutrophils, monocyte-derived macrophages, lymphocytes, and dendritic cells). The outcome following cardiac injury appears to depend largely on the number, kinetics, and phenotypes of these cells. Amongst them, innate immune cells, including monocytes, macrophages and neutrophils, have more established functions in clearing cell debris, propagating and resolving inflammation, and even promoting tissue restoration by secreting growth factors and remodeling the ECM [30]. On the other hand, the contribution of adaptive immune cells in cardiac repair and regeneration is slowly being revealed. For example, B cells are associated with autoimmunity against healthy CMs after cardiac injury [140], while T cells play versatile roles in autoreactivity, inflammation modulation and tuning macrophage polarity [122, 128, 141]. Undoubtedly, the question of cell-specific contributions to the overall injury response still requires extensive investigation. Here, we summarize information on a set of resident and recruited cells with more defined roles in modulating inflammatory response post cardiac injury.

#### Cardiac resident cells

##### Endothelial cells

As the most abundant cardiac resident cells, endothelial cells constitute > 60% of the non-myocytes in the mouse and zebrafish hearts [88, 90]. Endothelial activation is required for leukocyte extravasation during inflammation. For example, upon activation by DAMPs, P-selectin is significantly upregulated in endothelial cells allowing neutrophil adhesion and infiltration after I/R injury [130]. In addition to adhesive interactions, activated endothelial cells secrete cytokines and chemokines, including the monocyte chemoattractant protein-1 (MCP-1) to attract monocytes to the injured myocardium [57]. The roles of endothelial cells in immunity have recently been reviewed.

**Fig. 3:**
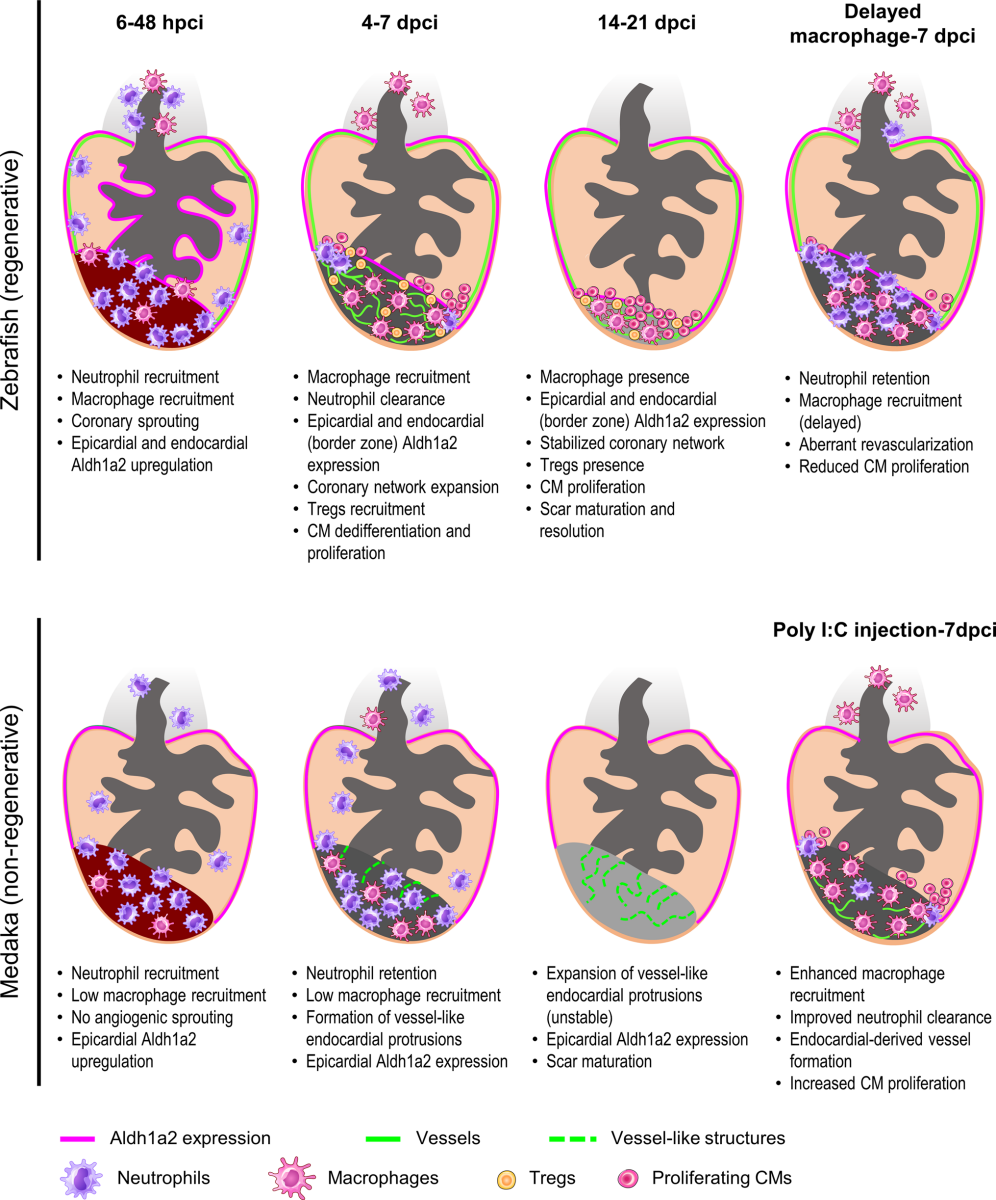
Comparative analyses in zebrafish and medaka after cardiac injury. At 6–48 h post cryoinjury (hpci) in zebrafish, neutrophils and macrophages have been recruited to the damaged tissue, coincident with angiogenic sprouting from existing coronaries and activation of *aldh1a2* expression in both the epicardium and endocardium. In medaka, we observed reduced macrophage recruitment compared to zebrafish, but similar neutrophil recruitment. Furthermore, medaka lacks both angiogenic sprouting and induction of endocardial *aldh1a2* expression during this period. At 4–7 days post cryoinjury (dpci) in zebrafish, neutrophils are gradually cleared by the increasing numbers of macrophages, while the coronary network expands to the whole injury area. Regulatory T cells (Tregs) are recruited to the damaged tissue and contribute to CM proliferation. On the other hand, in medaka, neutrophils are not cleared due to the reduced macrophage recruitment and remain in the injured area. Sporadic vessel-like structures formed by endocardial-derived cells appear at the border zone and there is no significant increase in CM proliferation. At 14–21 dpci in zebrafish, CMs actively proliferate and replace the collagen scar in a fully vascularized injured area. In medaka, vessel-like structures formed by the endocardial extensions are not stable and the collagen scar persists in the absence of replenishing CMs. Delayed macrophage recruitment in zebrafish, following pre-depletion, led to neutrophil retention, aberrant revascularization and reduced CM proliferation at 7 dpci. On the other hand, poly I:C-injected medaka exhibited enhanced macrophage recruitment and neutrophil clearance at 7 dpci, coincident with vessel formation and increased CM proliferation. How the immune response facilitates revascularization, CM dedifferentiation and proliferation, as well as scar resolution, seems to be key for a successful cardiac regeneration when comparing zebrafish to medaka

The cardiac endothelium is composed of endocardial and coronary endothelial cells, and both have been shown to be instrumental for heart regeneration. In zebrafish, early coronary invasion of the injured area is critical to support the regenerative response (Fig. 3) [65]. Such a fast coronary invasion, which has started by 15 h after cryoinjury in zebrafish, is not observed in non-regenerative models such as mice and medaka [65]. In addition, stable revascularization is dependent on timely macrophage recruitment, while delayed macrophage recruitment compromises revascularization (Fig. 3) [58]. Interestingly, while medaka lack a distinct coronary system, administration of the TLR3 agonist poly I:C can accelerate revascularization which is contributed by the endocardium in a macrophage-dependent manner (Fig. 3) [58]. These findings support a critical and instructive role of fast revascularization in accelerating cardiac regeneration. Correspondingly, accelerated revascularization after reperfusion coincided with higher inflammatory cell influx and better functional recovery when comparing I/R and permanent MI [123]. Despite the hints from these and other experimental studies, clinical attempts to promote revascularization through administration of different growth factors resulted in leaky and unstable blood vessels, revealing the urgent need to better understand the biology behind angiogenic revascularization. For example, combined approaches to stabilize new vessels by triggering pericyte/smooth muscle cell coverage might be essential for clinical therapeutics [76, 117]. Understanding the potential relationship between the immune response and coronary invasion/revascularization represents an important step in cardiac healing and requires further investigation.

##### Fibroblasts

Fibroblasts constitute the second largest population of cardiac resident cells in mouse, and they are usually quiescent and enmeshed in the interstitial tissue and perivascular matrix. Upon severe ischemia, interstitial fibroblasts serve as sentinels to detect myocardial injury and trigger inflammation [109]. DAMP-PRRs activation on fibroblasts leads to altered cellular function including changes in proliferation and migration, transdifferentiation into myofibroblasts, ECM turnover, and production of fibrotic and inflammatory paracrine factors [121]. In addition to DAMPs, ROS also stimulate inflammasome activation and IL-1b production in cardiac fibroblasts [51]. During this pro-inflammatory phase, cardiac fibroblasts are activated by DAMPs and ROS, and are maintained by IL-1 signaling. Later in the resolution/reparative phase, when the wound has been cleared by phagocytic cells and inflammation has been resolved, these fibroblasts transdifferentiate into myofibroblasts with a matrix synthetic function [105]. In non-regenerative models, cardiac repair following sudden loss of a large number of cardiomyocytes is dependent on the clearance of dead cells and the formation of a collagen-based scar to maintain structural integrity [109]. The active termination and the finely tuned fibrotic response of fibroblasts are, thus, critical to minimize hypertrophic remodeling and organ failure.

However, it remains unclear how fibroblasts contribute to heart regeneration, especially during scar resolution. Collagenous scar formation and maturation occur in regenerative models just as in non-regenerative models. However, scar resolution occurs only in regenerative models and it is dependent on fibroblast function. In salamanders, alternative activation of fibroblasts after macrophage depletion resulted in a permanent, highly cross-linked ECM scar and compromised heart regeneration. Similarly in zebrafish, fibroblasts appear to be required for scar resolution, as ablating *col1a2*-expressing fibroblasts impaired CM proliferation and scar resolution [102]. These data illustrate the critical and dynamic roles of fibroblasts in propagating inflammation and promoting scar formation and resolution during post infarct healing. Our understanding of the contribution, regulation and alternative activation of fibroblasts, as well as how fibroblasts interact with immune cells clearly requires more detailed studies.

##### Resident macrophages

**Fig. 4:**
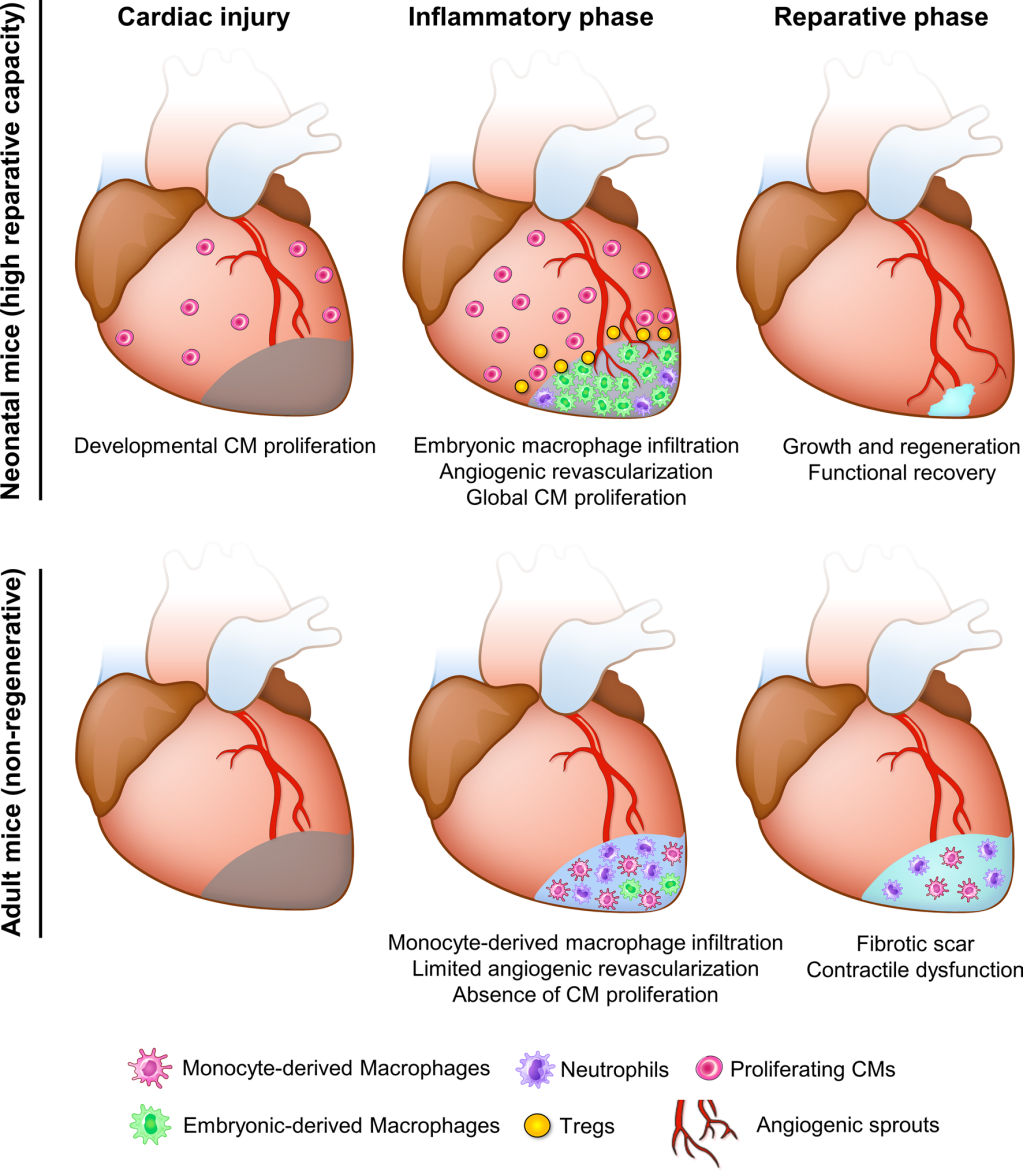
Comparative analyses in neonatal and adult mice after cardiac injury. In neonatal mice, embryonic macrophages with M2-like properties expand and dominate the injured area, leading to minimal inflammation, angiogenesis, and vigorous CM proliferation. T cells are also prone to differentiate into Tregs at this stage, resolving inflammation and stimulating CM proliferation by secreting mitogens [138]. The high reparative capacity leads to functional recovery in neonatal mice. However, in adult mice, this M2-like resident macrophage population is replaced, or out-numbered, by monocyte-derived macrophages which are prominently pro-inflammatory [59]. This functional difference leads to an unresolved scar and contractile dysfunction

Tissue-resident macrophages are the most abundant immune cell population in the heart, and they respond to damage signals by producing inflammatory cytokines and initiating neutrophil recruitment [89, 132]. Macrophages in adult mouse hearts constitute a heterogeneous population. The majority of cardiac resident macrophages derives from the yolk sac and is maintained in the heart through local proliferation [24]. These resident macrophages are distinct from mononuclear cells sorted from the spleen and brain of adult mice, and with a gene expression profile similar to anti-inflammatory M2 macrophages [91]. After genetic CM ablation in adult mice, this M2-like resident macrophage population was replaced, or out-numbered, by monocyte-derived macrophages which are prominently pro-inflammatory (Fig. 4) [59]. Furthermore, after systematic macrophage depletion, cardiac inflammation or aging, CCR2^+^Ly6C^hi^ monocytes replace embryonic-derived resident macrophages and coordinate cardiac inflammation [24, 74].

On the contrary, after MI in neonatal mice, the population of embryonic-derived resident macrophages which generates minimal inflammation expands and helps to mediate cardiac recovery by promoting angiogenesis and CM proliferation (Fig. 4) [59]. In neonatal mice, depletion of phagocytes (presumably resident macrophages) by clodronate liposomes compromised angiogenesis and regeneration [3]. Similarly, in zebrafish, depletion of macrophages by clodronate liposomes before cryoinjury led to defects in revascularization and CM proliferation at 7 dpci, despite the presence of macrophages (Fig. 3). These defects could be due to the loss of tissue-resident macrophages or a shift in macrophage polarity, which will be further discussed in the monocyte and macrophage section below.

#### Recruited inflammatory cells

##### Neutrophils

Neutrophils are rapidly recruited to injured tissues by DAMPs, cytokines and chemokines, activated complements, and histamine [70, 113]. They dominate the infarcted myocardium in the first 2 days post MI in adult mice [133], as well as post cardiac injury in zebrafish [58]. Corresponding to their well-established anti-pathogen function, neutrophils clear cell debris in the infarct, produce inflammatory cytokines, and accelerate monocyte influx [113]. Due to their pro-inflammatory and cytotoxic properties, excessive neutrophil activity has been associated with poor prognosis, remodeling and mortality after MI [1, 72].

In line with previous findings in adult mice, excess and prolonged neutrophil activity is largely associated with an unresolved inflammatory response, and potentially affects cardiac repair and regeneration. Ly6G^+^ neutrophil numbers recruited to the injured myocardium are reduced in neonatal mice compared to adults, which may lead to decreased pro-inflammatory cytokine production and decreased collateral tissue damage from neutrophil activity (Fig. 4) [3, 59]. In adult mice, insufficient removal of neutrophils resulting from decreased macrophage recruitment post MI leads to enhanced matrix degradation, delayed collagen deposition and increased susceptibility to cardiac rupture [62]. Comparative analyses of zebrafish and medaka revealed that neutrophil clearance after cardiac injury is delayed in the latter (Fig. 3). Similarly, neutrophil retention was associated with an excessive fibrotic response and unresolved scar upon macrophage depletion by clodronate liposomes in zebrafish (Fig. 3) [58].

On the beneficial side, neutrophils play an active role in the resolution of inflammation by secreting myeloperoxidase, which dampens the hydrogen peroxide burst after tissue wounding [87]. In addition, neutrophils orchestrate post-MI healing by polarizing macrophages towards a reparative phenotype [43]. Furthermore, neutrophils promote angiogenesis during inflammation by secreting VEGF [34], and their arrival in the injured area precedes the initiation of revascularization during zebrafish heart regeneration [65]. These findings suggest that therapeutic strategies to reduce acute inflammation driven by neutrophils after MI should be carefully considered as they might interfere with the healing response modulated by neutrophils including revascularization, macrophage polarization and resolution of inflammation. The potential roles played by neutrophils immediately after cardiac injury are particularly interesting and beg for further investigation in both regenerative and non-regenerative models.

##### Mononuclear phagocytes–monocytes and macrophages

Monocytes are a type of leukocytes which can differentiate into macrophages and dendritic cells. Monocyte-derived macrophages scavenge dead CMs, degrade their released macromolecules, and promote ECM remodeling and blood vessel formation [80]. Monocytes and monocyte-derived macrophages are functionally heterogenous [133]. Distinct Ly6C^hi^ and Ly6C^low^ monocyte subsets are sequentially recruited to injured hearts. Ly6C^hi^ monocytes peak during the early pro-inflammatory phase and exhibit phagocytic, proteolytic, and inflammatory functions, while Ly6C^low^ monocytes come later in the resolution phase, exhibit attenuated inflammatory properties, and express VEGF [80]. Differentially polarized M1- and M2-like macrophages are involved in biphasic repair processes (pro-inflammatory and resolution/repair phases) post MI [80, 110, 133]. In adult mice, CD206^+^F4/80^+^CD11b^+^ M2-like macrophages predominantly populate the infarct area and exhibit strong reparative abilities after MI, while their depletion resulted in a worsened prognosis and frequent cardiac rupture [110]. This phenomenon was due at least in part to impaired fibroblast activation and reduced collagen fibril formation [110]. In addition, this decreased tissue repair could be rescued by an external supply of M2-like macrophages, or an increase in M2-like macrophages by IL-4 administration [110]. Interestingly, M2 macrophages in the reparative phase of infarcted hearts seem to be derived from Ly6C^hi^ monocytes and proliferate locally to affect inflammation resolution and wound healing [41]. These data support the requirement of both Ly6C^hi^ and Ly6C^low^ monocyte subsets, and the beneficial effect of promoting M2 macrophage polarization during cardiac repair in adult mice.

The importance of macrophage polarization in heart regeneration has also been shown in zebrafish. Delayed macrophage recruitment by clodronate liposome pre-depletion compromised heart regeneration, despite control-like macrophage numbers in clodronate-treated hearts at 7 dpci (Fig. 3) [58]. Correspondingly, macrophage recruitment was also found to be delayed and significantly reduced in non-regenerative medaka post cardiac injury, and the kinetics and function of macrophage could be restored by poly I:C injections (Fig. 3) [58]. These findings indicate that macrophage polarization shifts during the regenerative response and the underlying mechanisms remain to be elucidated. As mentioned earlier, M2-like resident macrophages dominate in the neonatal mouse heart post MI and are essential for cardiac regeneration (Fig. 4) [59]. Together, these data support a role for macrophage polarization in modulating the inflammatory response and promoting heart regeneration.

Of note, the M1/M2 paradigm was initially based on the distinct function/phenotype of macrophage populations induced by a selected set of ligands [67]. Thus, the M1/M2 paradigm might be too simplistic in the context of tissue injury, as macrophages might constitute a broad spectrum of mixed phenotypes, an issue which requires significant further study [67]. The interactions between infiltrated macrophages and the complex and dynamic microenvironment of the cardiac infarct, the kinetics of macrophage polarization, and the potential macrophage phenotypes/functions await further investigation. Temporally coordinated differentiation of monocyte-derived M1 and M2 macrophages by distinct cytokines, DAMPs and other signaling molecules might accelerate cardiac healing and possibly even promote regeneration.

##### Lymphoid cells

Circulating lymphocytes comprise both B and T cells, which are the major cellular components of adaptive immunity. T cells are divided into CD8^+^ and CD4^+^ subsets: CD8^+^ T cells directly lyse target cells, while CD4^+^ T cells secrete various cytokines and orchestrate the immune response. Depending on their cytokine repertoire, CD4^+^ T cells are further classified as Th1 (IFN-γ, IL-2 and TNFα), Th2 (IL-4, IL-5, IL-13), Th17 (IL-17, IL-21, and IL-22) and regulatory T cells (Tregs) (TGF-β, IL-35). The significance of lymphocyte activation by autoantigens such as α-MHC released by the infarcted myocardium has only begun to be unraveled [42]. CD4^+^ T cell depleted mice, but not those lacking CD8^+^ T cells, exhibited significantly smaller infarcts than WT mice after I/R injury [135, 136]. Similarly, depletion of mature B lymphocytes impeded monocyte mobilization, limited myocardial injury and improved heart function post MI [140].

Among CD4^+^ T cells, Foxp3^+^CD25^+^ Tregs play a critical role in myocardial healing. Tregs enhance the recovery of damaged tissues by suppressing the immune response, promoting revascularization, and modulating monocyte/macrophage differentiation toward the M2 phenotype (Fig. 2) [128, 141]. In adult mice, expansion of Tregs by adoptive transfer or a CD28 superagonistic antibody attenuated the post-infarction inflammatory response, protecting the heart from adverse remodeling after MI [69, 118]. Clinical use of statin to decrease cholesterol levels after acute MI has also been shown to modulate the immune response by enhancing regulatory T-cell numbers and inhibiting pro-inflammatory T-cell subpopulations [28]. These lines of evidence suggest that Tregs are potent modulators of the immune response, which can limit and resolve inflammation, as well as attenuate ventricular remodeling, thereby improving cardiac function after MI.

It has been proposed that the mature and complex adaptive immune system in adult mammals compared to neonates and other evolutionarily more ancient animals (such as amphibians and fish) might be responsible for their limited regenerative capacity [33]. Consistently, immunomodulation aiming to restore cardiac tolerance was proposed to tune down adaptive immunity and accelerate regenerative therapies [103]. Comparison of CD4^+^ T cells in neonatal and adult mice revealed that T cells from neonates have an intrinsic “default” mechanism to become Tregs in response to T-cell receptor (TCR) stimulations, and that this ability gradually diminishes within the first 2 weeks after birth (Fig. 4) [126]. In line with this observation, the human fetal immune system generates more Tregs that suppress immune responses, especially autoantigen-specific immunity [75]. As first shown in zebrafish, Tregs are essential for heart regeneration (Fig. 3). Foxp3^+^ Treg-like cells (zTregs) home to damaged cardiac tissue starting from 3 days post injury and promote CM proliferation through the secretion of the CM mitogen Neuregulin [46]. Conditional ablation of zTregs impaired CM proliferation and heart regeneration [46]. In mouse, Tregs have been reported to promote CM proliferation during pregnancy, in both maternal and fetal hearts, as well as after MI in adults [138]. In addition, Tregs are indispensable for heart regeneration in neonatal mice, potentially by promoting monocyte/macrophage recruitment as well as CM proliferation (Fig. 4) [60] (a preprint article on biorxiv without peer-review). These data support an active role of Tregs in cardiac regeneration through immune modulation and the secretion of Neuregulin [46]. As promoting Treg function in cardiac repair may be therapeutically valuable, it will be important to reach a mechanistic understanding of this response.

### Resolution of inflammation

Timely resolution and containment of inflammation are critical for cardiac healing. Conversely, a prolonged inflammatory response leads to increased CM loss, cardiac remodeling, extensive fibrotic response, and even cardiac rupture [30]. From a comparative point of view, it is often speculated that the inflammatory response in non-regenerative models is excessive and prolonged after cardiac injury [104]. However, the precise spatial and temporal regulation of the inflammatory response toward scar-free regeneration after MI remains unclear. Compiling evidence puts macrophage function and polarization at center stage. Functionally heterogeneous M1 and M2 macrophages dominate the injured mouse heart at earlier (M1, 1-3 days) and later (M2, after 5 days) stages. Pro-inflammatory M1 macrophages express high level of pro-inflammatory cytokines, such as TNFα, IL-1 and IL-6; while reparative M2 macrophages express high level of anti-inflammatory cytokine IL-10, in addition to pro-inflammatory cytokines [133]. For a summary of macrophage phenotypes and their respective markers (mostly cytokines and chemokines) in mice, please see Kain et al. [50].

There have been several strategies proposed to inhibit post-MI inflammation, but early attempts have often resulted in adverse outcomes in clinical trials [38, 85]. On the other hand, approaches that focus on resolving post-MI inflammation have been sparsely used [50]. As an example, administration of the pro-resolving lipid mediator Resolving D in mice accelerated the inflammation resolution following MI and improved LV function [49]. Consistently, faster inflammatory resolution occurred after reperfusion, and coincided with increased inflammatory cell recruitment, accelerated revascularization, improved LV function and reduced remodeling [123]. These data suggest that facilitating active resolution may prevent persistent inflammation and heart failure. Strategies aiming to modulate inflammation sometimes lead to the opposite outcome (e.g., adverse remodeling vs. cardiac preconditioning/protection). This phenomenon is of great interest and worthy of systematic investigation to gain mechanistic insights. One hypothesis is that improved revascularization post reperfusion accelerates debris clearance, immune cell recruitment, turnover, and function, as well as inflammatory resolution, in a situation more similar to that in regenerative models (despite still not optimal enough for regeneration). Immune stimulation in a permanent MI model might achieve similar effects of an accelerated immune response and preconditioning/cardiac protection, but the same treatment might lead to over stimulation and/or unresolved inflammation in an I/R model. Of note, in both the comparisons between I/R injury and MI, as well as between zebrafish and medaka, an acute and robust immune response is associated with better functional outcome and is compatible with heart regeneration.

## Concluding remarks

Despite growing interest in the field, previous clinical trials that modulate inflammation after cardiac injury have been largely unsuccessful, partly due to the biphasic aspect of inflammation (lack of temporal control), as well as the general cytokines/immunosuppressors adopted (lack of specificity). Based on recent findings on the differential immune response between regenerative and non-regenerative models, modulating cellular processes exerting biphasic functions, including macrophage polarization and T-cell activation into Tregs, may represent a promising direction to promote cardiac healing and even regeneration. Understanding the potential interactions between immune cells (e.g., macrophages and Tregs) and between immune and non-immune cells (e.g., macrophages and fibroblasts) also requires more extensive studies (Fig. 2). In addition, the pleiotropic roles of TLR signaling represent another interesting aspect to explore in the context of MI, since activation of some TLR members seem to mediate immune modulation and preconditioning rather than conventional inflammation. In addition to the neonatal mouse model, where information directly relevant to regeneration is often mixed with the physiological changes of development, growth, and maturation [98], more information may be gained from non-mammalian models to further deepen our understanding of the permissive and instructive processes leading to heart regeneration. Clearly, complementary information from various models will greatly accelerate the understanding of endogenous regeneration and provide hints towards the development of effective therapies.
